# Valve-in-Valve TAVR in Surgical Stentless Aortic Bioprostheses, a Challenging Scenario

**DOI:** 10.3390/medicina62050844

**Published:** 2026-04-28

**Authors:** Sara Saltarocchi, Mizar D’Abramo, Emmanouela Chourda, Paolo De Orchi, Flaminia Spunticchia, Marco Totaro, Mattia Vinciguerra, Silvia Romiti, Gabriele Giunti, Ernesto Greco, Fabio Miraldi

**Affiliations:** 1 Department of General Surgery, Surgical Specialty and Anesthesiology “Paride Stefanini”, Sapienza University of Rome, 00161 Rome, Italy; 2 Cardiac Surgery Unit, Department of Cardiovascular Science, San Carlo Hospital, 85100 Potenza, Italy; 3 Internal, Clinical, Anesthesiological and Cardiovascular Sciences Department, Sapienza University of Rome, 00161 Rome, Italy; mizar.dabramo@uniroma1.it (M.D.); fabio.miraldi@uniroma1.it (F.M.); 4 Division of Cardiac Surgery, Azienda di Rilievo Nazionale e Alta Specializzazione “G. Brotzu”, 09134 Cagliari, Italy; 5 Department of Health and Life Sciences, European University of Rome, 00163 Rome, Italy; mattia.vinciguerra@unier.it (M.V.);

**Keywords:** TAVR, valve-in-valve, stentless, bioprosthesis, coronary obstruction

## Abstract

*Background and objectives*: Valve-in-valve transcatheter aortic valve replacement (ViV TAVR) has become an established treatment for failed surgical bioprostheses in patients at high surgical risk. However, procedures performed in degenerated stentless aortic valves remain particularly challenging because of the absence of a radiopaque frame, variable surgical implantation techniques, and a potentially increased risk of coronary obstruction. Evidence in this specific setting is limited. We conducted a systematic review of the literature to identify studies reporting ViV TAVI in degenerated stentless surgical bioprostheses. *Materials and methods*: Case reports and case series were included when patient-level or clearly identifiable data were available. Baseline characteristics, anatomical features, procedural strategies, and clinical outcomes were extracted and analyzed using a descriptive approach. A total of 54 studies were included, encompassing 294 ViV TAVI procedures performed in failed stentless aortic valves. *Results*: The mean patient age was 73.9 years, and the average STS-PROM score was 13.45%, reflecting a high-risk population. The most frequently treated prosthesis was the Medtronic Freestyle valve, and the predominant mechanism of failure was regurgitation. Transfemoral access represented the most common approach, while balloon-expandable and self-expanding transcatheter valves were used with similar frequency. Coronary protection strategies were adopted in a minority of procedures, whereas adjunctive procedural techniques such as pre- or post-dilation were relatively common. Device-related complications were mainly driven by coronary obstruction, while cardiac complications included myocardial infarction and unplanned coronary intervention. Overall, VARC-3 device success was achieved in the majority of procedures, with acceptable short-term mortality despite the complexity of the treated population. *Conclusions*: ViV TAVR in degenerated stentless bioprostheses appears feasible and generally effective but remains associated with specific procedural challenges, particularly related to coronary obstruction risk. Careful anatomical assessment and tailored procedural planning are essential, and larger contemporary studies are needed to better define optimal management strategies in this complex setting.

## 1. Introduction

Surgical aortic valve replacement (AVR) has long represented the standard treatment for patients with severe aortic valve disease. With the progressive aging of the population and the widespread use of bioprosthetic valves, structural valve deterioration has become an increasingly frequent clinical problem, often requiring reintervention years after the index procedure [1]. Over the last decade, transcatheter aortic valve replacement (TAVR) has profoundly changed the management of patients with degenerated surgical bioprostheses, offering a less invasive alternative to redo surgery in selected patients. In this context, valve-in-valve (ViV) TAVR has rapidly evolved from a bailout strategy into an established therapeutic option, with growing clinical experience and expanding indications [2,3].

Surgical stentless aortic bioprostheses were introduced with the aim of optimizing hemodynamic performance by eliminating the rigid stent frame, thereby providing a larger effective orifice area and lower transvalvular gradients compared with conventional stented valves, particularly in patients with small aortic roots. Over the past decades, several stentless valve designs have been widely implanted, including porcine root prostheses and subcoronary configurations, with initially favorable clinical and echocardiographic outcomes [4]. Despite these advantages, long-term durability of stentless bioprostheses remains limited, and structural valve deterioration (SVD) eventually occurs, manifesting as aortic regurgitation, stenosis, or mixed valve dysfunction. Redo surgical aortic valve replacement in patients with failed stentless prostheses is technically challenging due to extensive adhesions, altered root anatomy, and the frequent need for complex root reconstruction. Consequently, redo surgery in this population is associated with increased operative risk, particularly in elderly patients and those with multiple comorbidities [5].

**Figure 1:**
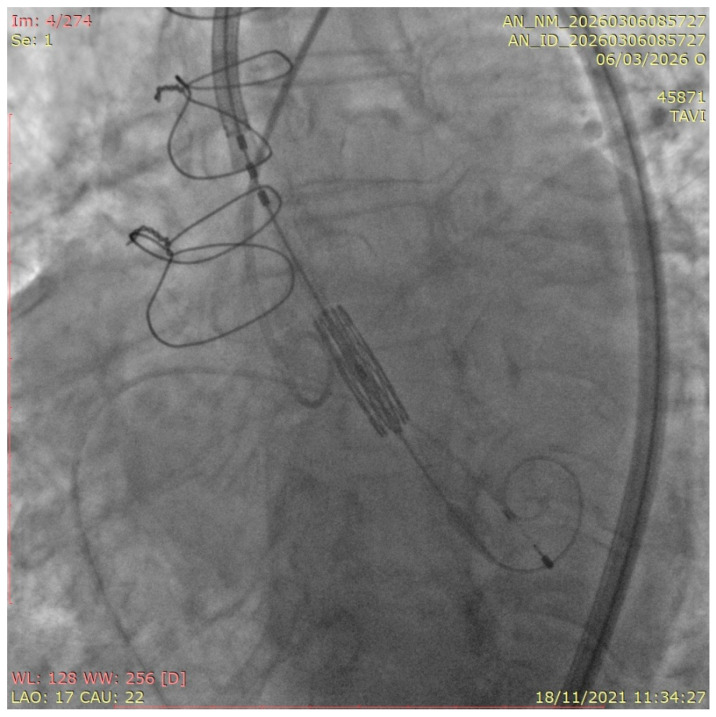
ViV TAVR in a Freestyle bioprosthesis. Pre-deployment of a Sapien 3 THV in a stentless Freestyle valve; note the absence of fluoroscopic and anatomical landmarks.

**Figure 2:**
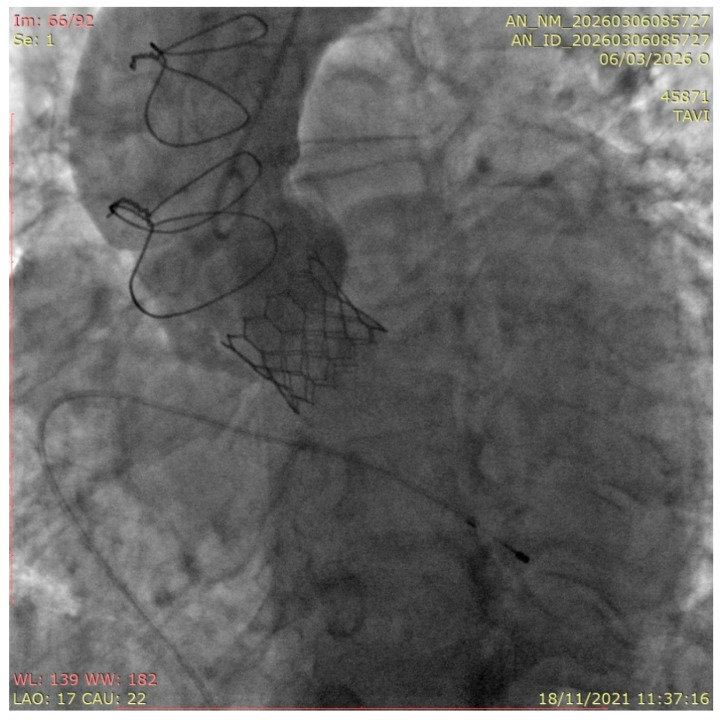
ViV TAVR in a Freestyle bioprosthesis. Result of the procedure with a fully deployed Sapien 3 THV in a Freestyle valve with optimal positioning and no complications.

Transcatheter valve-in-valve replacement has emerged as an effective alternative to redo surgery for failed surgical bioprostheses and is now an established treatment option in selected high- and intermediate-risk patients. However, most of the evidence supporting ViV TAVR has been derived from studies predominantly including stented surgical valves. In contrast, stentless bioprostheses represent a distinct anatomical and procedural setting, characterized by the absence of a rigid frame, variable leaflet insertion techniques, and a close spatial relationship between valve leaflets and coronary ostia (Figure 1 and Figure 2).

**Figure 3:**
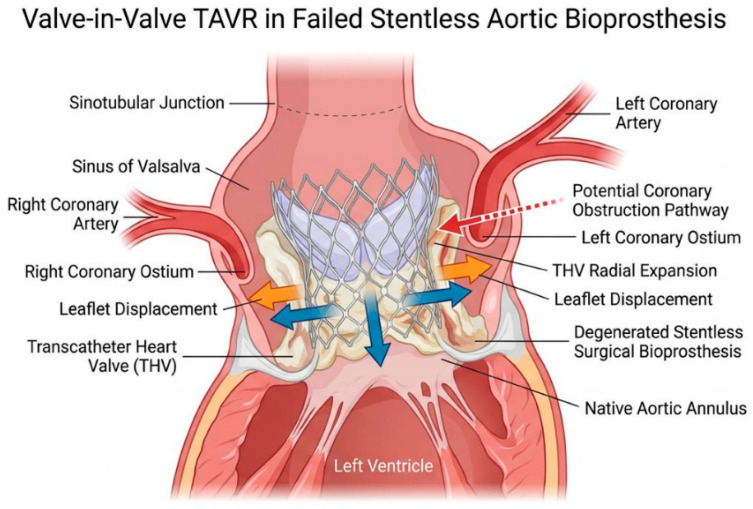
Overview of anatomical features and technical challenges in ViV procedures in stentless bioprostheses.

These anatomical features pose unique challenges during ViV TAVR in stentless valves, including difficulties in accurate valve sizing and positioning, limited fluoroscopic landmarks, and a markedly increased risk of coronary artery obstruction or valve embolization (Figure 3). As a result, ViV TAVR in stentless prostheses is frequently considered a high-risk procedure, often requiring advanced preprocedural planning, multimodality imaging, and adjunctive strategies such as pre- or post-dilation, coronary protection or chimney stenting in selected cases.

Despite the clinical relevance of this scenario, available evidence on ViV TAVR in stentless valves remains fragmented and heterogeneous. Published data consist largely of small case series, multicenter registries, and individual case reports, often with inconsistent reporting of valve types, mechanisms of failure, procedural strategies, and clinical outcomes. Furthermore, stentless valves are frequently underrepresented or pooled together with stented prostheses in larger ViV registries, limiting the ability to draw valve-specific and population-specific conclusions.

Given these limitations, a comprehensive synthesis of the existing literature focusing specifically on ViV TAVR in surgical stentless aortic bioprostheses is warranted. The present systematic review aims to summarize patient characteristics, mechanisms of stentless valve failure, procedural approaches, and early clinical outcomes associated with ViV TAVR in this challenging subset, with the goal of providing practical insights to inform procedural planning and clinical decision-making.

## 2. Methods

**Figure 4:**
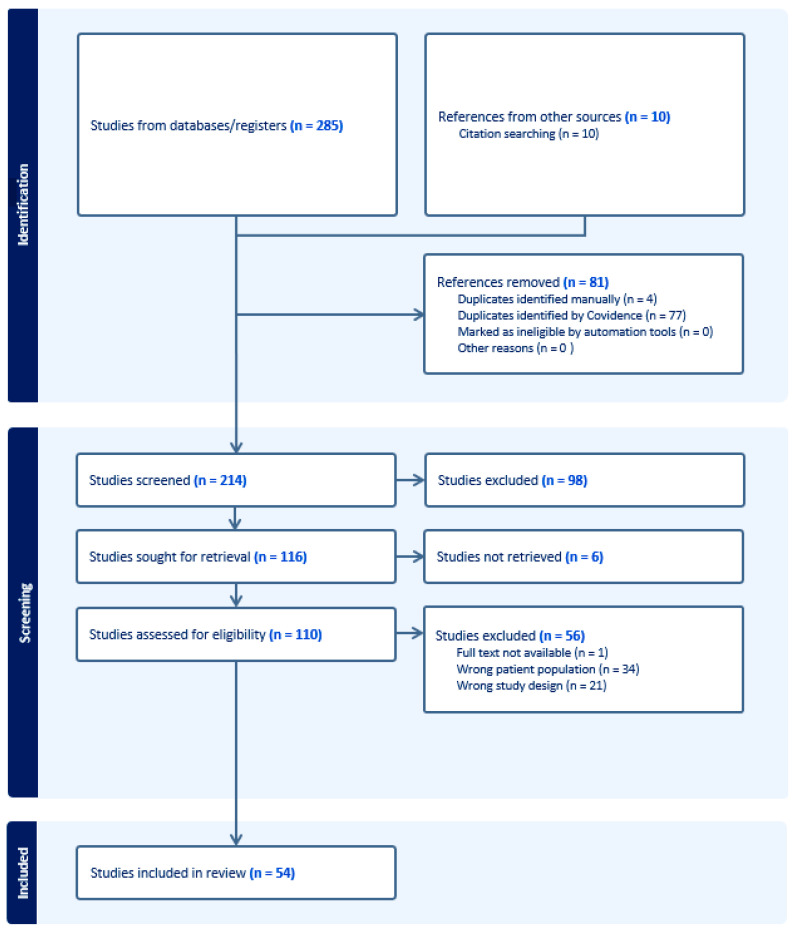
PRISMA flow diagram.

A systematic review of the literature was conducted to identify studies reporting valve-in-valve TAVR in patients with failed surgical stentless aortic bioprostheses. The review was performed in accordance with the Preferred Reporting Items for Systematic Reviews and Meta-Analyses (PRISMA) recommendations; the PRISMA 2020 checklist is provided in the Supplementary Material (Supplementary Figure S1) [6]. The research question was defined according to the PICO framework. The population of interest included adult patients with degenerated surgical stentless aortic bioprostheses; the intervention was ViV TAVR; no formal comparator was prespecified; outcomes of interest included procedural characteristics and early clinical outcomes following ViV TAVR (Figure 4). A comprehensive literature search was performed in the PubMed, Embase, and Scopus databases from inception to 2026/10/01, using a combination of free-text terms related to valve-in-valve procedures and stentless aortic valves, including “valve-in-valve”, “TAVI”, “TAVR”, “stentless”, and the names of commonly used stentless bioprostheses (e.g., “Freestyle”,” Toronto”, “Freedom”). Reference lists of selected articles were manually screened to identify additional relevant studies. The full electronic search strategies for each database are reported in the Supplementary Material.

Study selection was independently performed by two investigators (SS and MDA), who screened titles and abstracts and subsequently assessed full-text articles for eligibility. Disagreements were resolved by consensus. Studies of any design (including case reports, case series, single center observational studies and multicenter registries) were considered eligible if they reported ViV TAVR procedures performed in surgical stentless aortic bioprostheses and provided identifiable, extractable clinical or procedural data. Studies including mixed populations of stented and stentless bioprostheses were excluded when stentless-specific data could not be separated. Reviews, editorials, and conference abstracts without original data were excluded. For studies reporting mixed populations, only patients with stentless surgical valves and available individual-level data were included. Each ViV TAVR procedure performed in a stentless bioprosthesis was considered an independent case. In one collaborative registry, relevant inconsistencies between patient-level tabulated data and the descriptive text on in-hospital outcomes were identified. Given the inability to resolve these discrepancies, despite attempts to contact the authors, the study was excluded from the review to preserve data integrity. Additionally, articles in which the physiological context differed substantially from other included studies (for example, procedures performed in the setting of a ventricular assist device with altered hemodynamics) were also excluded.

Data were extracted by SS and MDA using a predefined data collection sheet, including patient characteristics, type of stentless bioprosthesis, echocardiographic features, mechanism of valve failure, procedural strategy and details, use of coronary protection techniques, complications, and early clinical outcomes. Data collection and management were performed using Microsoft Excel (Microsoft Corporation, Redmond, WA, USA).

Procedural success and clinical outcomes were recorded according to Valve Academic Research Consortium (VARC)-3 definitions when explicitly reported by the original studies. When VARC criteria were not specified, outcomes were collected as defined by the authors.

The literature search identified 54 studies meeting the predefined inclusion criteria, published between 2010 and 2025. The majority of included publications consisted of case reports and small case series, reflecting the rarity and clinical complexity of valve-in-valve TAVR in failed stentless aortic bioprostheses. Only a limited number of studies reported multicenter experiences or registry-based data. Overall, a total of 294 ViV TAVR procedures performed in surgical stentless bioprostheses were included in the analysis. Given the marked heterogeneity of study designs, populations, and reported outcomes, results are presented using a descriptive and qualitative approach, while maintaining a systematic methodology.

All included studies are summarized in Table 1, along with their most relevant features and outcomes, i.e., type of stentless valve, type of TAVR, mechanism of valve failure, coronary protection or adjunctive strategies reported, main complications and outcomes in terms of mortality and procedural success. These features will be more deeply discussed in the results section. For studies that were not case reports, the number of patients listed in the study-level table refers exclusively to those patients meeting the inclusion criteria for this review. In some publications reporting a broader series of ViV procedures, only a subset of procedures involved stentless valves and were therefore extracted and analyzed. In the study by Chourdakis et al. [7], more than one ViV procedure is described for the same patient. For consistency, we considered only the first ViV, which was performed in a stentless valve, as subsequent procedures occurred in a non-stentless setting.

Device success according to Valve Academic Research Consortium version 3 (VARC-3) [60] was either directly reported by the authors or calculated based on available procedural and outcome data, when feasible. Although hemodynamic variables such as post-procedural gradient and prosthesis–patient mismatch are important components of VARC definitions, these were not uniformly available in all studies. When post-procedural echocardiographic data were explicitly reported, device success was derived (“D” in Table 1) based on this information. In the absence of reported data, VARC-3 device success was not mentioned.

Adjunctive procedural strategies were defined as additional maneuvers performed to optimize valve deployment or mitigate specific anatomical risks, including pre-dilation, post-dilation, leaflet modification techniques (e.g., BASILICA), valve fracturing, and other intra-procedural optimization approaches.

In this first outline, complications were categorized as device-related or cardiac as follows. Device-related complications included events such as device embolization, maldeployment, and coronary obstruction. Cardiac complications included ventricular perforation, annular rupture, cardiac tamponade, aortic dissection, and cardiac arrest. In some instances, a single clinical event could be reasonably classified in both categories (for example, left main coronary artery obstruction with subsequent cardiac arrest that required mechanical circulatory support), and these were recorded accordingly in the study-level table.

In a small number of reports, ViV procedures were initiated but not completed due to intra-procedural events that prevented successful implantation. These cases were identified and noted in the dataset but were not included in analyses of successful ViV outcomes unless clearly stated otherwise.

Given the heterogeneity of study designs and the predominance of case reports and small case series, results were synthesized descriptively, and no formal meta-analysis was planned.

Moreover, we performed a temporal subgroup analysis to account for the rapid evolution of transcatheter aortic valve implantation (TAVI) technology and procedural strategies over time. Early studies largely reflect the use of first-generation devices and limited operator experience, whereas more recent reports incorporate newer-generation transcatheter heart valves and more refined procedural planning. On this basis, studies were stratified according to publication year (≤2017 vs. ≥2018) to explore potential differences in outcomes related to these advancements. This cut-off was chosen considering the 2017 ACC Expert Consensus Decision Pathway for TAVR as a key point in TAVR patients’ evaluation, since it provided a framework for managing a potential TAVR candidate from indication to post-TAVR management [61]. Comparison of procedural and clinical outcomes between studies published up to 2017 and those published from 2018 onwards was made using Fisher’s exact test.

The methodological quality of the included studies was assessed qualitatively using the Joanna Briggs Institute (JBI) critical appraisal tools for case reports and case series, when applicable. Most studies fulfilled 6–8 JBI criteria, indicating overall low risk of bias. Specific JBI tables for case reports and case series are reported in Supplementary Material (Supplementary Tables S1 and S2).

## 3. Results

### 3.1. Baseline and Anatomical Characteristics

Baseline patient characteristics were reported with variable completeness across studies. An overview is offered in Table 2.

Mean age was 73.9. Demographic data, including age and sex, were available in most reports, as well as clinical presentation, whereas comorbidities were seldom described, the main focus being chronic obstructive pulmonary disease (COPD), previous stroke or transient ischemic attack, coronary artery disease and chronic kidney disease (CKD). For some of these baseline variables, particularly in case reports, the absence of mention could not be reliably interpreted as either true absence of the condition or lack of reporting. Because this distinction could not be consistently established across studies, the “studies reporting” column was not applied to these variables.

Information on surgical risk was often provided (33 studies), with risk scores such as Society of Thoracic Surgeons Predicted Risk of Mortality (STS-PROM) score or EuroSCORE II reported in most studies. Mean STS-PROM score among included studies was 13.45%. Critical preoperative state was defined as the presence of cardiogenic shock, need for inotropic or mechanical circulatory support, or procedures performed under urgent/emergent conditions.

A wide range of surgical stentless bioprostheses were represented and reported in 51 studies and 216 patients. Medtronic Freestyle^TM^ valves were the predominant prostheses, present in 146 patients (67.5%), although other models, like Freedom Solo^TM^ (17.5%), Toronto SPV^TM^ (6%), Freedom Solo^TM^, Shelhigh^TM^ (3.2%), Cryolife-O’Brien^TM^ (0.9%), 3f Enable^TM^ (0.9%), Prima Plus^TM^ (0.9%), BioValsalva^TM^ (0.9%),Tissuemed^TM^ (0.4%), Biocor^TM^ (0.4%), Solo Smart^TM^ (0.4%) and Pericarbon Freedom^TM^ (0.4%) were also described. In a minority of cases (three studies, 76 patients), the exact type of stentless prosthesis was not specified.

Prosthesis failure, largely attributed to SVD, was mainly due to valve regurgitation (62.7%), while valve stenosis or combined mechanisms were present in a minority of cases (21.7% and 15.5%, respectively). However, the degree of detail used to describe valve degeneration varied considerably across reports, with some focusing primarily on clinical presentation rather than echocardiographic findings. Indeed, preoperative echocardiographic assessment was inconsistently detailed. Left ventricular systolic function, as well as mean transvalvular gradients, were seldom reported, while comprehensive anatomical measurements were available only in a few cases.

Anatomical features potentially influencing procedural complexity, such as coronary ostial height, sinus dimensions, or previous aortic root interventions, were selectively described. High risk coronary anatomy, defined as a right and/or left coronary artery (RCA, LCA) height distance of ≤12 mm between the aortic annulus plane and the coronary ostia or a valve-to-coronary (VTC) distance ≤ 4 mm, identified via CT scan, was present in 53 patients but reported in only 16 studies.

### 3.2. Procedural Characteristics

A summary of procedural characteristics is provided in Table 3. Procedural characteristics were analyzed descriptively, including access route, device type, use of adjunctive strategies, and coronary protection measures. Given the heterogeneity in reporting across studies, the number of studies contributing data for each variable is specified, allowing patient-level percentages to be interpreted in the context of available evidence. Access route was almost always reported (46 studies, 274 patients). Transfemoral access was the most commonly reported approach when specified (61.3%), followed by transapical access (10.9%), transaxillary (1.8%) and transcarotid (1%). Nine studies, including a total of 68 patients, did not specify the typology of access; therefore, complete data regarding this matter are available only for 75.2% of patients. Both balloon-expandable (46.7%) and self-expanding (52.2%) transcatheter heart valves were employed, while mechanically expandable valves were utilized only on two occasions (1%). Preventive strategies aimed at mitigating the risk of coronary obstruction were described in a subset of patients (40 studies, 35 patients), particularly in those with high-risk anatomical features. Among them, protection with guidewires or undeployed stents was performed in 24 (68.5%) patients; chimney/snorkel stenting was performed in seven patients (20%), while BASILICA was performed in four patients (11.4%). Information regarding adjunctive procedural steps, including balloon pre-dilation and post-dilation, with the purpose of limiting potential TAVR malpositioning and embolization, was variably reported (38 studies, 92 patients): in 46.7% of cases, a balloon pre-dilation was needed, while in 53.2% a post-dilation was performed.

### 3.3. Complications and Outcomes

Procedural and clinical outcomes and complications were largely reported, though with heterogeneous definitions and variable levels of detail across studies. Device success according to Valve Academic Research Consortium 3 criteria was reported in a substantial proportion of cases and derived the remnant cases.

Device-related complications occurred globally in 38 patients (12.9%), the main complication being coronary obstruction (23 patients), followed by TAVR maldeployment (12 patients) and embolization (three patients). Cardiac complications happened in 21 cases (7.1%). Myocardial infarction was considered when periprocedural acute ischemic manifestations were reported, such as ST-segment elevation, new ischemic ECG changes, ventricular arrhythmias, hemodynamic instability, or cardiac arrest in the setting of coronary compromise. This happened in 13 patients, typically following coronary obstruction after THV deployment. In this setting, unplanned PCI of the left main coronary artery was necessary in four cases. Mitral valve damage during TAVI delivery and ventricular perforation with guidewires occurred, respectively, in just one case. In two cases, unexplained cardiac arrest or worsening of left ventricular function occurred in the perioperative period without any specified cause. A second valve deployment was necessary in five cases (1.7%). Major vascular complications happened in 19 patients (6.5%), neurological events in seven patients (2.4%), and acute kidney injury in 18 patients (6.1%). Permanent PMK was implanted in 14 patients (4.8%). Major bleeding occurred in 34 patients (11.6%). Mechanical support with ECMO, IABP or Impella was necessary in eight patients (2.7%), as well as conversion to open surgery. Patient–prosthesis mismatch, defined as a postoperative echocardiographic mean pressure gradient ≥ 20 mmHg, was present in 10 patients (3.4%), while significant (at least moderate) aortic regurgitation was present in six patients (2%). Minor complications were rarely detailed.

As already stated, device success according to Valve Academic Research Consortium version 3 (VARC-3) [60] was either directly reported by the authors or calculated based on available procedural and outcome data when feasible. In this review, only four studies included enough data for VARC-3 device success calculation. Our analysis evidenced a VARC-3-defined device success rate of 62.6% (184 patients), while overall 30-day mortality was 7.48% (22 patients). It is important to highlight that this result must be read with caution, since not every study includes VARC-3 device success analysis or the criteria necessary for its validation. If we reframe our analysis including only the studies that reported VARC-3 data (directly or calculated), we find a more encouraging 77.3% (184 patients from a population of 238 patients).

Minor complications were rarely detailed. A summary of early procedural and clinical outcomes is presented in Table 4.

### 3.4. Temporal Analysis

Temporal trends in complications and outcomes are shown in Table 5. This analysis was performed only on available data of Table 1 and demonstrated a significantly higher use of adjunctive strategies in more recent studies (37.4% vs. 18.5%, *p* < 0.0001), reflecting a more proactive and preventive procedural approach. In parallel, a significant reduction in cardiac complications (15.9% vs. 4.8%, *p* = 0.008) and of device-related complications (23.8% vs. 10.0%, *p* = 0.013) was observed, suggesting improved procedural safety over time. Conversely, no significant differences were found in the rates of coronary protection, procedural success according to VARC-3 criteria, or 30-day mortality.

## 4. Discussion

Valve-in-valve TAVR in failed surgical stentless aortic bioprostheses represents one of the most technically demanding subsets of transcatheter aortic interventions. The present review, encompassing 294 procedures across 54 studies, confirms that this clinical scenario is characterized by high surgical risk, complex anatomy, and non-negligible procedural hazards, yet acceptable technical success and outcomes when performed in experienced centers [2,62].

Patients undergoing ViV in stentless prostheses were elderly and predominantly symptomatic, with more than half presenting in advanced NYHA class. The mean STS-PROM score was markedly elevated, underscoring the high operative risk profile that justifies a transcatheter strategy in most cases. Comorbidities such as coronary artery disease, chronic kidney disease, and COPD were frequent, reflecting a frail population in whom redo surgery would carry substantial morbidity and mortality. Importantly, a small subset of patients presented in a critical preoperative state, including cardiogenic shock or need for inotropic or mechanical support. Although numerically limited, this group highlights how ViV TAVR in stentless valves is often undertaken as a bailout or urgent strategy rather than as an elective structural degeneration procedure [33].

Unlike stented bioprostheses, where structural degeneration often leads to stenosis, failed stentless valves in this cohort most commonly presented with predominant regurgitation [4]. This observation is clinically relevant: pure regurgitation in a stentless root may reflect leaflet degeneration without a rigid sewing ring or frame to guide anchoring, thereby increasing procedural complexity. The absence of a radiopaque stent frame and the potential distortion of the aortic root further complicate fluoroscopic positioning [63]. Despite the predominance of regurgitant failure—typically considered a more favorable substrate for valve expansion—the incidence of device-related complications remained notable, particularly for coronary obstruction. However, no clear relationship was observed between failure mode and procedural outcomes in our cohort, suggesting that anatomical factors inherent to stentless prostheses may play a more dominant role than the mode of degeneration itself. Moreover, a significant proportion of patients were classified as having high-risk coronary anatomy based on MDCT criteria. In the stentless setting, coronary obstruction risk is influenced not only by ostial height but also by sinus dimensions, sinotubular junction geometry, leaflet length, and virtual transcatheter valve-to-coronary distance. This multifactorial anatomical interplay explains why coronary obstruction emerged as the most frequent device-related complication in the present analysis [64].

Transfemoral access was the dominant approach, reflecting contemporary TAVR practice, even if, in our analysis in almost 25% of cases, there was no indication of the selected access. However, the non-negligible use of transapical access likely reflects earlier experience and anatomical constraints in complex redo scenarios. Device selection was relatively balanced between balloon-expandable and self-expanding valves. This suggests that no single platform has emerged as clearly superior in this setting, and that operator preference and anatomical considerations drive choice. Balloon-expandable valves may offer more controlled deployment in regurgitant anatomies but could increase radial stress, whereas self-expanding devices may better conform to distorted roots and be recaptured but require careful depth control to avoid delayed coronary compromise and pacemaker implantation [64,65]. Pre- and post-dilation were frequently employed, likely reflecting the need to optimize anchoring in the absence of a rigid frame. Coronary protection strategies were used selectively, predominantly with guidewire-based protection and, less frequently, chimney stenting or BASILICA. The fact that protection was used in a minority of cases despite a measurable proportion of high-risk anatomy suggests either under-recognition in early experience or a tailored strategy reserved for anatomically extreme cases.

The relatively low rate of permanent pacemaker implantation should be interpreted with caution, as pre-existing pacemaker status was not consistently reported, limiting accurate assessment of new implantations. However, we must consider that the optimal TAVI anchoring location in a stentless valve is at the noncompliant suture ring [66]. For example, in the Freestyle valve, which is the one more represented in our analysis, the fabric sewing cuff is sutured to the native annulus; therefore, ViV location may be at a different distance from the conduction tissue compared to the TAVR, which may explain the relatively low rates of PMK implantation in this series.

The most striking procedural signal in this review is the rate of coronary obstruction, which represented the leading complication. Established risk factors for coronary obstruction include low coronary ostial height (historically defined as <10 mm, although many contemporary studies consider < 12 mm as a higher-risk threshold), a short virtual transcatheter valve-to-coronary distance (VTC < 4 mm, with 4–6 mm considered borderline), small sinus of Valsalva diameter (generally < 30 mm), and a narrow or low sinotubular junction (sinotubular junction diameter ≤ 30 mm or narrower than the sinus diameter). In the valve-in-valve setting, the risk is further increased by outward displacement of the failed surgical bioprosthetic leaflets toward the coronary ostia, particularly in the absence of a rigid supporting frame as in stentless prostheses [64]. ViV in stentless valves carries a coronary risk profile that may exceed that of standard ViV in stented bioprostheses; the reported frequency is about 3.5% in ViV procedures and 1% in native valve TAVI [2], while in our analysis, ViV in stentless valve was associated with a 7.8% incidence of coronary obstruction (23 patients/294). Among cases of coronary obstruction, 52.2% (12 out of 23 patients) had undergone coronary protection. The relatively low overall use of this strategy, despite its application in approximately half of the patients who developed obstruction, suggests a selective and tailored approach, likely reserved for anatomically more complex or higher-risk cases. This may reflect evolving operator experience and risk recognition rather than systematic application, with coronary protection being preferentially employed in the highest-risk anatomies. Given the non-negligible overall incidence of coronary obstruction in this cohort (7.8%), a systematic consideration of coronary protection may be warranted, particularly in anatomically complex cases. Coronary protection strategies were used predominantly with guidewire-based protection and, less frequently, chimney stenting or Bioprosthetic Aortic Scaffold Intention-guided Laceration (BASILICA). In this context, advanced preventive strategies include chimney/snorkel stenting and BASILICA. Chimney stenting involves placing a coronary stent from the ostium into the aorta alongside the transcatheter valve to maintain coronary flow, and has shown safety and feasibility in retrospective series; however, concerns remain regarding long-term stent durability, coronary reaccess, prolonged dual antiplatelet therapy, and potential stent deformation during future interventions [67]. BASILICA represents a more physiological approach, consisting of intentional leaflet laceration to create a channel for coronary flow, with experimental data suggesting it may reduce sinus and neosinus flow stagnation. In lower-risk cases, a simple guidewire or undeployed stent can be utilized.

Importantly, not all coronary obstructions translated into clinically overt myocardial infarction, reflecting the impact of immediate percutaneous management or pre-emptive protection. Nevertheless, periprocedural myocardial infarction, ventricular arrhythmias, and hemodynamic collapse were observed and occasionally required mechanical circulatory support. These findings emphasize that coronary events in this setting can be rapidly catastrophic, with reported mortality around 50% in some series [67]. Second valve deployment, conversion to surgery, and need for mechanical support further illustrate the procedural fragility of this population. Although numerically limited, these events carry significant prognostic implications and highlight the importance of meticulous pre-procedural planning [65].

Meticulous planning should be considered also to predict patient–prosthesis mismatch (PPM). As stated, our analysis evidenced an incidence of PPM of 3.4%. Prosthesis–patient mismatch occurs when the effective orifice area is too small in relation to patient body size. Several studies have reported that PPM in surgically replaced aortic valves is frequent, with severe PPM ranging from 2 to 20%, while moderate PPM is reported as high as 70% in some cohorts [68]. The PARTNER TRIAL (COHORT A) described an incidence of PPM of 60.0% (severe: 28.1%) in the surgical cohort versus 46.4% (severe: 19.7%) in the TAVI cohort [69].

In ViV, the dimensions of the surgically implanted prosthetic valve, and therefore a pre-existing PPM, deeply affect post-operative results [70]: in this study, 7.6% of patients had preoperative PPM, and this population reached a higher incidence of post-ViV PPM (47.6%) compared to non-PPM patients (29.5%), with an increased risk of mortality at 1 year; in another study, small aortic annuli were associated with an increased rate of PPM (59.2%) compared to non-small annuli (44.4%) in patients undergoing ViV in mostly stented bioprostheses [71]. Similar results were obtained also in the VIVID study [72], where a strong association between the size of the valve and the incidence of PPM was highlighted. Our results, which evidenced an importantly lower incidence of PPM (3.4%), must be evaluated carefully, since, as already stated, our population evidenced a prevalence of the Freestyle bioprosthesis, used mostly in aortic root replacement settings, where generally the aortic valve annulus in “non-small”: in fact, in our cohort, surgically implanted prosthesis size range between 21 to 31, with a mean size of 25.4, and this may explain the low incidence of PPM in this analysis.

Despite anatomical complexity, VARC-3 device success was achieved in the majority of cases. As already stated, not every study in this review includes VARC-3 device success analysis or the criteria necessary for its validation; therefore, those results must be read with caution. However, our analysis evidenced a VARC-3-defined device success rate ranging from 62.6% to 77.3%. This result, even if not conclusive, highlights that the incidence of significant complications, prosthesis–patient mismatch and moderate or greater residual aortic regurgitation was relatively low, suggesting that acceptable hemodynamic performance can be achieved even in the absence of a surgical stent frame. This finding is reassuring and supports the feasibility of the transcatheter approach in experienced hands. However, the heterogeneity in reporting of post-procedural gradients across studies limits definitive conclusions regarding systematic hemodynamic optimization.

Thirty-day mortality, while not negligible, appears consistent with the high-risk profile of the treated population. Compared with ViV TAVI in stented valves, as analyzed in the PARTNER 2 Valve-in-Valve Registry [73], 30-day all cause mortality was significantly higher (7.48% vs. 2.7%), and this findings is far more significant if we consider that, in the PARTNER 2 ViV registry, mortality fell from the initial value of 8.2% to 0.7% (mean 2.7%), due to the learning curve of the procedure. Similar findings did not appear in our analysis: since most of the cited studies were monocentric and often case report/case series, there was no evidence of a complex learning curve in the ViV-in-stentless-valve procedure. The absence of a real learning curve, associated with the advanced age, comorbidity burden, and occasional critical presentation of the patients in this review, may explain this difference. In many cases, ViV TAVI likely represented the only viable therapeutic option.

One of the possible limitations in this analysis may be the temporal heterogeneity of the included studies: the timeframe starting from 2010 encompasses a period of substantial evolution in TAVI technology, including significant changes in valve design, implantation techniques, and patient selection, all of which directly impact procedural safety and outcomes; moreover, important developments in imaging (particularly CT-based planning), coronary protection strategies (e.g., BASILICA), and operator experience have occurred. Overall, the findings on temporal analysis (reduction in cardiac and device-related complications and widespread use of adjunctive strategies) support the hypothesis that advancements in device technology and increasing operator experience have translated into improved procedural management and reduced complication rates, although their impact on short-term mortality remains limited, likely due to the low event rate.

Taken together, the data suggest that although ViV in stentless valves is technically demanding and associated with meaningful procedural risk—particularly coronary complications—early survival outcomes are satisfactory considering the clinical context.

Several practical considerations emerge from this analysis. Meticulous MDCT planning is mandatory, with systematic assessment of coronary height, sinus dimensions, VTC, and leaflet length. Coronary protection strategies should be liberally considered in high-risk anatomies, especially in the absence of a surgical frame. Center experience likely plays a critical role, given the need for rapid bailout PCI, mechanical support, or surgical conversion. Device selection should be individualized, as current evidence does not clearly favor one platform over another.

### Future Directions

The available evidence remains limited by small sample sizes, retrospective design, and heterogeneity in reporting. Several areas warrant further investigation: prospective registries dedicated specifically to ViV in stentless valves; standardized MDCT-based risk stratification models for coronary obstruction in this subset; comparative analyses between balloon-expandable and self-expanding platforms in stentless anatomies; long-term durability and hemodynamic performance beyond early follow-up; and the role of systematic coronary protection or prophylactic leaflet modification strategies in anatomically high-risk patients.

## 5. Limitations

Most of the studies included in our analysis were case reports/case series. As already stated in the discussion, this may be a cause for concern in the absence of a described learning curve: favorable first cases and dramatic complications, as well as impressive bailouts, may be more suitable material for publication compared to uneventful procedures, with either good results or uncomplicated failures. Only a few studies described the experience of the center in more than 50 procedures; therefore, no proper analysis of experienced centers could be performed. Furthermore, there was substantial heterogeneity in the key variables reported, including echocardiographic and anatomical parameters, such that performing a structured, comparative analysis was difficult, if not impossible. Those limitations should be considered in the interpretation of our results. To overcome these limitations, an international registry of ViV in stentless valves should be evaluated, in order to find the best strategy to achieve optimal ViV in those high-risk patients.

## 6. Conclusions

Valve-in-valve TAVR in failed surgical stentless bioprostheses is a feasible but technically demanding procedure performed in a predominantly high-risk and symptomatic population. While overall device success and early survival are acceptable, the incidence of coronary obstruction and other procedure-related complications underscores the critical importance of meticulous pre-procedural imaging and individualized procedural planning. Coronary anatomy assessment, appropriate device selection, and readiness for adjunctive or bailout strategies are central to optimizing outcomes in this complex setting.

## Figures and Tables

**Table 1:** Main features of included studies.

Author	Year	Study Design	Number of Patients	Stentless Valve	Mode of Failure	THV Type	Coronary Protection (*n*)	Adjunctive Strategies (*n*)	Cardiac Complications (*n*)	Device-Related Complications (*n*)	VARC-3 Device Success	30-Day Mortality
Allende et al. [8]	2014	Case Report	1	FSB	R	BEV	1	/	1	1	0	0
Arunothayaraj et al. [9]	2021	Case Series	1	FSB	R	SEV	/	0	0	0	1	0
Bagur et al. [10]	2011	Case Report	1	FSB	S	BEV	/	/	0	0	1	0
Bapat et al. [11]	2014	Case Series	4	T-SPV, FSB, CLOB, PF	/	BEV	3	0	1	0	3	0
Basman et al. [12]	2023	Case Report	1	3FE	/	SEV	1	/	0	0	1 (D)	0
Baudo et al. [13]	2025	Single Center	15	FSB, T-SPV, PP, 3FE	3 S, 2 M, 8 R	9 BEV, 6 SEV	3	5	0	1	12	1
Bauernschmitt et al. [14]	2020	Case Report	1	FSB	R	BEV	/	/	0	0	1	0
Burgdorf et al. [15]	2021	Case Series	27	FSB	R	BEV	/	3	1	0	24	3
Burmeister et al. [16]	2025	Case Report	1	/	R	/	/	/	0	1	0	0
Chakravarty et al. [17]	2013	Case Report	1	T-SPV	R	BEV	1	0	1	1	0	0
Chatfield et al. [18]	2021	Case Series	1	FSB	R	BEV	0	0	0	0	1	1
Chevalier et al. [19]	2014	Case Report	1	FSB	M	BEV	0	1	0	0	0	0
Chourdakis et al. [7]	2025	Case Report	1	FSB	R	SEV	1	0	1	1	0	0
Cockburn et al. [20]	2017	Case Series	6	FS	3 S, 2 M, 1 R	2 BEV, 1 MEV, 2 SEV	1	1	2	2	4	1
Conzelmann et al. [21]	2018	Prospective	2	FSB, TM	/	SEV	0	/	2	0	0	2
Dallan et al. [22]	2020	Case Report	1	FSB	R	SEV	1	/	1	1	0	0
D’Ancona et al. [23]	2012	Case Report	1	FSB	R	BEV	1	/	0	0	/	0
Elzanaty et al. [24]	2022	Case Report	1	FSB	R	BEV	/	/	0	0	1 (D)	0
Fairley et al. [25]	2014	Case Report	1	T-SPV	R	SEV	0	0	0	0	1	0
Fiorina et al. [26]	2012	Case Series	1	CLOB	R	SEV	0	0	1	1	0	0
Gara Ali et al. [27]	2020	Case Report	1	FSB	R	SEV	0	0	0	0	1	0
Gonska et al. [28]	2016	Single Center	2	FSB	1 S, 1 R	BEV	0	0	0	0	1	0
Greif et al. [29]	2012	Case Series	1	FSB	S	BEV	0	1	0	0	1	0
Grubitzsch et al. [5]	2017	Single Center	27	/	/	12 SEV, 15 BEV	0	5	4	7	/	5
Gustafson et al. [30]	2023	Case Report	1	FSB	R	BEV	0	1	0	0	1	0
Halapas et al. [31]	2014	Case Report	1	FS	R	SEV	0	0	0	0	1 (D)	0
Hamudi et al. [32]	2018	Case Report	1	FSB	R	SEV	0	0	0	0	/	0
Hanson et al. [33]	2018	Case Report	1	FSB	R	BEV	0	0	0	0	1	0
Harmel et al. [34]	2017	Case Report	1	FSB	R	SEV	0	0	0	1	0	0
Huber et al. [35]	2015	Case Series	3	SH	R	SEV	/	/	0	0	3	0
Ielasi et al. [36]	2021	Case Report	1	T-SPV	S	BEV	1	0	0	0	/	0
Kanamori et al. [37]	2021	Case Report	1	FSB	R	BEV	0	0	1	1	0	0
Kapetanakis et al. [38]	2011	Case Report	1	SH	S	BEV	0	1	0	0	1	0
Karimi et al. [39]	2017	Case Report	1	FSB	M	BEV	1	0	0	0	1	0
Luc et al. [40]	2016	Case Report	1	FSB	R	BEV	0	1	0	1	0	0
Maggio et al. [41]	2017	Case Report	1	FS	S	BEV	1	0	0	0	/	0
Nagendran et al. [42]	2015	Case Report	1	FSB	R	SEV	1	0	0	0	1	0
Olusan et al. [43]	2025	Case Series	3	SH	1 M, 2 R	SEV	1	2	0	0	2	0
Rayner et al. [44]	2025	Multicenter	65	FS, FSB, BV, T-SPV	17 S, 14 M, 34 R	45 SEV, 20 BEV	14	25	0	7	60 *	0
Rodés-Cabau et al. [45]	2010	Case Report	1	FSB	R	BEV	0	0	0	0	1	0
Ruparelia et al. [46]	2017	Case Series	1	FSB	M	MEV	0	0	0	0	0	0
Sang et al. [47]	2018	Single Center	22	FSB	2 S, 2 M, 18 R	SEV	0	4	0	6	16	0
Sarkar et al. [48]	2011	Case Report	1	B-PSV	R	SEV	/	/	0	0	1	0
Schneider et al. [49]	2019	Single Center	8	FSB	6 S, 2 M	6 BEV, 2 SEV	1	0	3	2	6	2
Sharp et al. [50]	2010	Case Report	1	FSB	R	BEV	/	/	0	0	1	0
Sponga et al. [51]	2017	Case Report	1	FS	R	SEV	1	0	0	0	1	0
Steul et al. [52]	2025	Multicenter	43	/	17 S, 12 M, 14 R	37 SEV, 6 BEV	1	36	2	0	29	3
Takei et al. [53]	2023	Case Report	1	FSB	R	BEV	/	/	0	0	1	0
Tamura et al. [54]	2023	Case Report	1	SS	R	BEV	0	1	0	0	1 (D)	0
Taniguchi et al. [55]	2021	Case Report	1	FSB	R	BEV	0	1	0	1	0	0
Vukadinovkij et al. [56]	2021	Single Center	25	FSB	R	/	/	4	0	0	/	4
Yamashita et al. [57]	2019	Single Center	3	FSB	1 S, 2 M	2 BEV, 1 SEV	/	/	0	2	1	0
Yong et al. [58]	2014	Case Report	1	T-SPV	R	SEV	/	/	0	1	1	0
Zhong et al. [59]	2020	Case Report	1	FSB	S	SEV	/	/	0	0	1	0

THV: Transcatheter heart valve. FSB: Freestyle; T-SPV: Toronto; CLOB: Cryolife-O’Brien; PF: Pericarbon Freedom; 3FE: 3f Enable; PP: Prima Plus; FS: Freedom Solo; TM: Tissuemed; SH: Shelhigh; BV: BioValsalva; B-PSV: Biocor; SS: Solo Smart. S: stenosis; M: mixed; R: regurgitation. BEV: balloon-expandable valve. SEV: self-expandable valve. MEV: mechanically expandable. D: derived. * The article reports 60 cases of VARC-3-defined technical success. However, three cases of moderate aortic insufficiency are also described. It is unclear whether these cases are included among the five patients already excluded from the analysis or whether the number of device successes should instead be considered 57.

**Table 2:** Baseline and anatomical characteristics. TIA: transient ischemic attack. NYHA: New York Heart Association. COPD: chronic obstructive pulmonary disease. CKD: chronic kidney disease. LCA: left coronary artery. RCA: right coronary artery. VTC: valve-to-coronary.

Baseline Characteristics (Patients *n* = 294; Studies *n* = 54)		Studies Reporting (*n*)
**Age, mean**	73.9	53
**Sex**	171 M, 89 F	51
**STS-PROM score**, mean	13.45%	33
**COPD**, *n* (%)	48 (16.3%)	/
**CKD**, *n* (%)	55 (18.7%)	/
**Previous stroke/TIA**, *n* (%)	28 (9.5%)	/
**Extracardiac arteriopathy**, *n* (%)	42 (14.2%)	/
**Coronary artery disease**, *n* (%)	96 (32.8%)	/
**NYHA class ≥ III**	165 (56.1%)	/
**Critical preoperative state**, *n* (%)	19 (6.46%)	/
**Type of stentless prosthesis**, *n* (%)	**216**	51
Freestyle	146 (67.5%)	
Toronto SPV	13 (6%)	
Cryolife-O’Brien	2 (0.9%)	
Shelhigh	7 (3.2%)	
Pericarbon Freedom	1 (0.4%)	
Freedom Solo	38 (17.5%)	
Solo Smart	1 (0.4%)	
3f Enable	2 (0.9%)	
Prima Plus	2 (0.9%)	
Biocor	1 (0.4%)	
Tissuemed	1 (0.4%)	
BioValsalva	2 (0.9%)	
**Mode of failure**, *n* (%)	**258**	50
Stenosis	56 (21.7%)	
Mixed	40 (15.5%)	
Regurgitation	162 (62.7%)	
**High-risk coronary anatomy**, *n* (LCA/RCA height ≤ 12 mm or VTC ≤ 4 mm)	**53**	16

**Table 3:** Procedural characteristics. THV: transcatheter heart valve. BASILICA: Bioprosthetic Aortic Scaffold Intention-guided Laceration.

Procedural Characteristics		Studies Reporting (*n*)
**Access route**, *n* (%)	**274**	46
Transfemoral	168 (61.3%)	
Transapical	30 (10.9%)	
Transaxillary	5 (1.8%)	
Transcarotid	3 (1%)	
**Type of THV**, *n* (%)	**199**	49
Balloon-expandable valve	93 (46.7%)	
Self-expanding valve	104 (52.2%)	
Mechanically expandable valve	2 (1%)	
**Coronary protection**, *n* (%)	**35**	40
Guidewire or undeployed stent	24 (68.5%)	
Chimney/snorkel	7 (20%)	
BASILICA	4 (11.4%)	
**Adjunctive strategies**, *n* (%)	**92**	38
Pre-dilation	43 (46.7%)	
Post-dilation	49 (53.2%)	

**Table 4:** Complications and outcomes. PMK: pacemaker. PCI: percutaneous coronary intervention. LM: left main. RCA: right coronary artery. PPM: patient–prosthesis mismatch.

Outcome, *n* (%)	
**Device-related complications**	**38 (12.9%)**
Embolization	3
Coronary obstruction	23
Maldeployment	12
**Cardiac complications**	**21 (7.1%)**
Ventricular perforation	1
Mitral valve damage	1
Myocardial Infarction	13
Unplanned PCI	4
LM	4
RCA	0
Unexplained cardiac arrest/shock	2
**Vascular complications (major)**	**19 (6.5%)**
**Neurological complications**	**7 (2.4%)**
**Acute kidney injury**	**18 (6.1%)**
**Permanent PMK**	**14 (4.8%)**
**Major bleeding**	**34 (11.6%)**
**2nd valve deployment**	**5 (1.7%)**
**Mechanical support**	**8 (2.7%)**
**Conversion to surgery**	**8 (2.7%)**
**VARC-3 device success/overall**	**184/294 (62.6%)**
**VARC-3 device success/available data**	**184/238 (77.3%)**
**PPM (PG mean ≥ 20 mmHg)**	**10 (3.4%)**
**At least moderate aortic regurgitation**	**6 (2%)**
**30-day mortality**	**22 (7.48%)**

**Table 5:** Temporal trends analysis for complications and outcomes before and after 2017.

Outcome, *n* (%)	Population Tot = 63 ≤2017, *n* (%)	Population Tot = 231 >2017, *n* (%)	*p*-Value
Coronary protection	11/56 (19.6%)	24/170 (14.1%)	0.39
Adjunctive strategies	10/54 (18.5%)	82/219 (37.4%)	<0.0001
Cardiac complications	10/63 (15.9%)	11/231 (4.8%)	0.008
Device-related complications	15/63 (23.8%)	23/231 (10%)	0.013
VARC-3 device success	23/34 (67.6%)	161/204 (78.9%)	0.14
30-day mortality	6/63 (9.5%)	16/231 (6.9%)	0.58

## Data Availability

No new data were created or analyzed in this study.

## Supplementary Materials

The following supporting information can be downloaded at: https://www.mdpi.com/article/10.3390/medicina62050844/s1, Figure S1: PRISMA 2020 Checklist for systematic reviews; Table S1: JBI critical appraisal tool for Case Reports; Table S2: JBI critical appraisal tool for Case Series; electronic search strategies.

## Abbreviations

AVR	aortic valve replacement
ViV	valve-in-valve
TAVR	transcatheter aortic valve replacement
SVD	structural valve deterioration
PRISMA	Preferred Reporting Items for Systematic Reviews and Meta-Analyses
VARC	Valve Academic Research Consortium
COPD	chronic obstructive pulmonary disease
CKD	chronic kidney disease
STS-PROM	Society of Thoracic Surgeons Predicted Risk of Mortality
BASILICA	Bioprosthetic Aortic Scaffold Intention-guided Laceration
PCI	percutaneous coronary intervention
RCA	right coronary artery
LCA	left coronary artery
PPM	patient–prosthesis mismatch
VTC	valve-to-coronary
TIA	transient ischemic attack
NYHA	New York Heart Association
